# Progressive Medicine, Volume III

**Published:** 1900-01

**Authors:** 


PROGRESSIVE MEDICINE, VOLUME III.—A Quarterly Digest of Advances, Discoveries and Improvements in the Medical and Surgical Sciences. Edited by Hobart Amory Hare, M. D., Professor of Therapeutics and Materia Medica in the Jefferson Medical College of Philadelphia. Octavo, handsomely bound in cloth, 440 pages, 11 illustrations. Lea Brothers & Co., Philadelphia and New York.

This volume of Progressive Medicine considers the following subjects:

"Diseases of the Thorax and its Viscera, Including the Heart, Lungs, and Blood-vessels." By William Ewant, M. D., F. R. C. P., Physician and Joint Lecturer on Medicine, St. George's Hospital, London.

"Diseases of the Skin." By Henry W. Stelwagon, M. D., Clinic Professor of Diseases of the Skin, Jefferson Medical College, Philadelphia.

"Diseases of the Nervous System." By William G. Spiller, M. D., Professor of the Diseases of the Nervous System in the Philadelphia Polyclinic.

"Obstetrics." By Richard C. Norris, M. D., Instructor in Obstetrics, University of Pennsylvania, Philadelphia.

This volume, like Volumes I and II, gives in original narrative form a full and clear statement of all practical advances made in the departments discussed during the year. All the volumes are handsomely bound and finely illustrated. At the subscription price "Progressive Medicine" offers a splendid investment to any physician who is endeavoring to keep step in the procession.

